# Dementia Coding, Workup, and Treatment in the VA New England Healthcare System

**DOI:** 10.1155/2014/821894

**Published:** 2014-02-19

**Authors:** Kelly Cho, David R. Gagnon, Jane A. Driver, Arman Altincatal, Nicole Kosik, Stephan Lanes, Elizabeth V. Lawler

**Affiliations:** ^1^Massachusetts Veterans Epidemiology Research and Information Center, VA Boston Healthcare System, Boston, MA, USA; ^2^Division of Aging, Department of Medicine, Brigham and Women's Hospital, Harvard Medical School, 150 S. Huntington Avenue, Boston, MA 02130, USA; ^3^Boston University School of Public Health, Boston, MA, USA; ^4^New England Geriatric Research Education and Clinical Center, VA Boston Healthcare System, Boston, MA, USA; ^5^United BioSource Corporation, Lexington, MA, USA

## Abstract

Growing evidence suggests that Alzheimer's disease and other types of dementia are underdiagnosed and poorly documented. In our study, we describe patterns of dementia coding and treatment in the Veteran's Administration New England Healthcare System. We conducted a retrospective cohort study with new outpatient ICD-9 codes for several types of dementia between 2002 and 2009. We examined healthcare utilization, medication use, initial dementia diagnoses, and changes in diagnoses over time by provider type. 8,999 veterans received new dementia diagnoses during the study period. Only 18.3% received a code for cognitive impairment other than dementia, most often “memory loss” (65.2%) prior to dementia diagnosis. Two-thirds of patients received their initial code from a PCP. The etiology of dementia was often never specified by ICD-9 code, even by specialists. Patients followed up exclusively by PCPs had lower rates of neuroimaging and were less likely to receive dementia medication. Emergency room visits and hospitalizations were frequent in all patients but highest in those seen by dementia specialists. Dementia medications are commonly used off-label. Our results suggest that, for the majority the patients, no prodrome of the dementia syndrome is documented with diagnostic code, and patients who do not see dementia specialists have less extensive diagnostic assessment and treatment.

## 1. Introduction

Approximately 13% of Americans over age 65 have Alzheimer's disease (AD), the most common cause of dementia [1]. This prevalence estimate comes from carefully conducted population-based studies and is substantially higher than that seen in healthcare settings. The prevalence of all types of dementia among users of the U.S. Department of Veterans Affairs Healthcare System (VAHCS) aged 55 years and older, as defined by ICD9 diagnosis codes, is 7.3% [2]. Growing evidence suggests that AD and other types of dementia are under-diagnosed and poorly documented in most primary care settings, particularly in their early stages [3, 4].

While cognitive screening in asymptomatic individuals is not widely recommended [5], there is a growing appreciation of the importance of early recognition of dementia. Patients with unrecognized impairment do not get tested for reversible causes of dementia, do not get counseling regarding the disease process or advanced care planning, and are not offered treatment. Undiagnosed cognitive impairment can compromise patient safety, medication compliance, and patient-doctor communication [3, 6].

Integrated Service Network 1 (VISN1), the New England region of VAHCS, serves over 230,000 patients. To better understand patterns of dementia coding and treatment in this population, we assembled a retrospective cohort of VISN1 patients with the three most common dementia diagnoses. Our goals were to describe patterns of diagnostic coding and treatment by provider type and healthcare utilization patterns around the diagnostic period.

## 2. Methods

Data for this study were extracted from the VA National Patient Care and Decision Support Systems Databases and the clinical, laboratory, and pharmacy files of VISN1 data system. This study was reviewed and approved by the Institutional Review Board and Research and Development Committees of the VA Boston Healthcare System.

All subjects in VISN1 with healthcare service between January 1, 2002, and December 31, 2009, who received an outpatient ICD-9 code for dementia of the Alzheimer's type (331.0, 290.0–290.3; DAT), vascular dementia (290.4–290.43; VD), or dementia not otherwise specified (294.8; DNOS) from a PCP, neurologist, geriatrician, psychiatrist, psychologist, or neuropsychologist were eligible for study inclusion. Cognitive impairment was defined as any of the following codes: (1) mild cognitive impairment (MCI, 331.83), (2) memory loss (780.93), (3) late cognitive effects of cerebrovascular disease (438.0, CDCVD), and (4) cognitive disorder not otherwise specified (CDNOS, 294.9). All patients had to be at least 55 years old at the time of first dementia diagnosis. A one-year baseline period prior to first dementia diagnosis was used to evaluate prior history of resource utilization and medication use. ICD-9 codes for cognitive impairment were allowed during the 12 months prior to a recorded dementia diagnosis. Individuals were followed for one year after the date of first dementia diagnosis, unless this was preceded by either their last visit to the VA Healthcare System, death, or the calendar end of the study (December 31, 2009). The one-year period before and after first dementia diagnosis is referred to as the “peridiagnostic period.”

We defined the initial diagnosis as the first code for cognitive impairment. We identified all occurrences of diagnoses for dementia or cognitive impairment during the study period. We defined the final diagnosis as follows: (1) patients were considered to have a final diagnosis of DAT or VD if they had at least one relevant code and no codes for other specific types of dementia (DNOS was permitted); (2) patients with codes for both AD and VD in the absence of any other specific dementia code were considered to have a final diagnosis of mixed dementia; (3) patients with one or more codes for DNOS and no specific codes for dementia or Parkinson's disease were classified as DNOS. We identified the type of provider to give the initial cognitive diagnosis (PCP, mental health provider, neuropsychologist, geriatrician, neurologist, and other providers). We also identified the type of provider patients seen in the year before and after diagnosis and created the following categories: (1) followed by PCPs only; (2) ever seen by neurologists or geriatricians; (3) ever seen by mental health providers (psychiatrists or psychologists).

### 2.1. Statistical Analyses

We report summary statistics (frequencies and proportions for categorical variables and means for continuous data) to describe diagnostic patterns by provider type, baseline characteristics of dementia patients, and resource utilization. We examined the first and final dementia diagnoses by provider type. We described overall resource utilization rates in the peri-diagnostic period (1 year before and after diagnosis). Resource utilization included visits to the emergency room (ER) and hospitalizations and use of neuroimaging and medications (dementia medications, antipsychotics, and antidepressants). Comorbidities were defined by outpatient ICD-9 codes and imaging studies by CPT codes. We used the VISN1 pharmacy file to describe the use of dementia medications (cholinesterase inhibitors and memantine), antipsychotics, and antidepressants. All statistical analyses were performed using SAS (Statistical Analysis Software) 9.2.

## 3. Results

**Figure 1:**
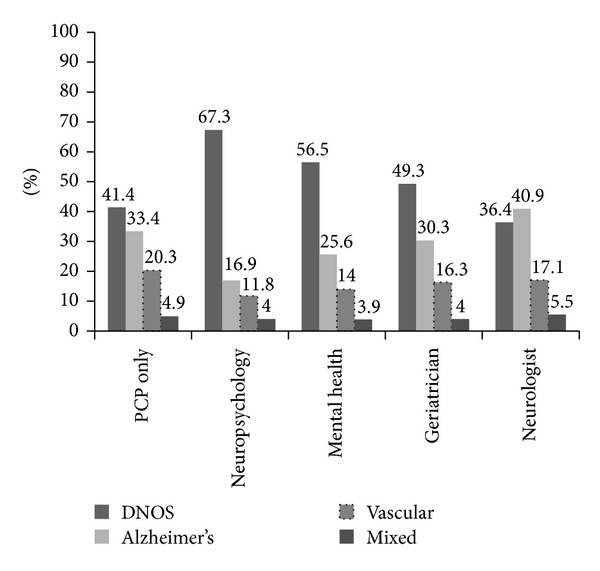
Distribution of final dementia diagnoses by provider category.

We identified 8,999 subjects who met study inclusion criteria (Table 1). Only 1,643 (18.3%) patients received a diagnosis for a prodromal cognitive syndrome prior to their initial dementia code. Among patients with cognitive impairment, memory loss was the term most frequently assigned (65.2%), followed by CDNOS (29.7%). MCI (2.7%) was rarely used as an initial diagnosis. Among patients initially diagnosed with dementia, DNOS (41.9%) was the most common initial dementia diagnosis, followed by DAT (25.0%) and VD (14.9%). During the peri-diagnostic period, 26.9% of patients were seen by neurologists or geriatricians, 34.1% by mental health specialists, and 39.1% by PCPs only. PCPs documented the initial dementia code for 66.5% of patients (*n* = 5,998), followed by specialists without expertise in dementia (9.3%), mental health providers (8.2%), and neurologists (7.7%). The most frequent initial dementia diagnosis given by neurologists was DAT; for all other provider types DNOS was most frequently used. The mean age at first dementia code of this predominantly male cohort was 79.5 years. Patients followed exclusively by PCPs had the oldest mean age (80.2 yrs), while those seen by psychiatrists and psychologists were younger (76.5 yrs) and had more comorbidity than other groups. The most common final dementia diagnosis was DNOS (44.3%), followed by DAT (31.9%), VD (18.9%), and mixed DAT/vascular (4.8%). The distribution of final diagnoses by physician type is displayed in Figure 1. The initial dementia diagnosis was changed in less than 30% of patients who were followed by PCPs only, while among those seen by neurologists and geriatricians 40.8% of initial AD diagnoses, 79% of initial VD diagnoses, and 64.4% of initial DNOS diagnoses were changed.

**Figure 2:**
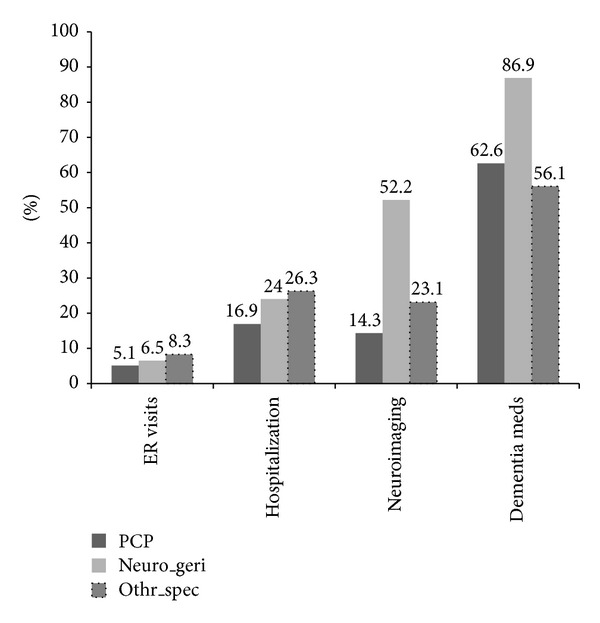
Resource utilization during peridiagnostic period by provider type one year before and after first dementia diagnosis.

Figure 2 summarizes healthcare utilization in the peri-diagnostic period. Rates of ER visits and hospitalization were highest in patients seen by psychiatrists and psychologists (26.3%) or neurologists and geriatricians (24.0%) and were lower in those seen by PCPs only (16.9%). Imaging rates were much higher among patients seen by neurologists and geriatricians (52.2%) than by other dementia specialists (23.1%) and PCPs (14.3%). Dementia medications were prescribed in 66.8% of patients with a final diagnosis of DAT, 27.7% of patients with VD only, and 65.7% of patients with DNOS only. The rate of dementia medication use was 86.9% in those seen by neurologists or geriatricians, 62.6% in those followed up only by PCPs, and 56.1% in those seen by the other dementia specialists.

## 4. Discussion

Dementia is a chronic, slowly progressive condition whose diagnosis is frequently missed by patients, family members and medical providers. In this retrospective cohort study among veterans diagnosed with dementia in the VA New England Healthcare System, we found that few patients are given a diagnosis of cognitive impairment prior to their first dementia diagnosis. This is despite the fact that many of them have been followed up in the system for years. The majority of patients receive their first dementia diagnosis from a PCP. Compared to patients who see a geriatrician or neurologist, dementia patients followed up exclusively by PCPs are less likely to receive a specific dementia diagnosis and less likely to have their initial diagnosis change over time. They are also less likely to have neuroimaging or receive dementia medication. The rates of emergency room care and hospitalization were substantial in the year before and after initial dementia diagnosis. Together, these findings suggest that, for the majority of VA patients, dementia is likely not diagnosed at an early stage, and patients who do not see dementia specialists have less extensive diagnostic assessment and treatment.

Less than 20% of the patients in our cohort had a code for cognitive impairment prior to dementia. Underreporting of cognitive impairment in primary care is an important cause of delay in the diagnosis of DAT, a dementia for which there is approved treatment that may benefit even those with early dementia [7, 8]. Accurate diagnosis of dementia, especially by PCPs, is an ongoing challenge. A European study of dementia diagnosis in primary care found that clinicians identified dementia by clinical judgment 73% of the time but only documented the diagnosis for 38% of their patients [9]. Severity of cognitive impairment seems to be a major predictor of documentation. According to a recent meta-analysis of 8 studies in a non-VA setting, diagnostic sensitivity for dementia by PCPs was only 49% [10]. Over 60% of patients with severe dementia were diagnosed, as compared to 9–41% of those with early dementia. Overall, the findings of this study suggest that about half of the types of dementia go undiagnosed in the primary care setting. Benefits of an earlier diagnosis include the opportunity for education, management of neuropsychiatric symptoms, avoidance of medications that worsen cognitive function, improved medication safety and compliance, and help with support services and future planning.

This study highlights the overuse of nonspecific dementia diagnoses by VISN1 providers but reflects a much wider trend both within and outside the VA [2, 11]. PCPs infrequently changed the initial dementia diagnosis over time, while geriatricians and neurologists frequently changed DNOS to a specific diagnosis. Neuropsychologists were the most likely of all providers to use a nonspecific diagnosis, perhaps because they defer making a definitive diagnosis to the provider who ordered the testing. Neurologists and geriatricians were substantially more likely to obtain neuroimaging than PCPs. Neurologists were the most likely to provide patients with a specific dementia diagnosis. It is possible that more complicated patients are referred to specialists, but this would not account for the high prevalence of nonspecific diagnoses by PCPs. The obstacles to dementia diagnosis in primary care are well documented and include important systems issues such as time constraints and lack of support services [12]. New models of dementia diagnosis and care are needed that base resources in the primary care setting.

The lack of emphasis on a more accurate diagnosis of dementia is linked to the absence of effective treatment options. Active screening in older adults will be clearly justified once disease-modifying therapy is available. Specialized neuroimaging and fluid biomarkers, now mostly used in the research setting, are viable approaches to detecting early and even preclinical AD [13, 14]. With emerging evidence of genetic risk factors for sporadic AD, such as apolipoprotein E (APOE) *ε*4 allele [15, 16], genotyping may also become incorporated into routine algorithms for screening and diagnosis.

Rates of ER visits and hospitalizations in the peri-diagnostic period were substantial in all patients but notably higher among patients seen by dementia specialists. One explanation could be that dementia is often recognized in the setting of acute cognitive or behavioral problems that necessitate emergency care, prompting referral to specialists. An important and unanswered question is whether earlier diagnosis and management of cognitive impairment in the primary care setting could help decrease morbidity and costs associated with this increased resource utilization [17, 18].

Cholinesterase inhibitors and memantine are FDA approved for the treatment of AD, for which they have been shown to provide modest benefits. These drugs are not approved for mild cognitive impairment or other types of dementia, although they may have efficacy in dementia with Lewy bodies [19]. In this study, about two-thirds of patients with a final diagnosis of AD were prescribed a dementia medication in the peri-diagnostic period, while about one-third of patients with a code for VD only or DNOS also received dementia medication. VA formulary criteria restrict the use of cholinesterase inhibitors to patients with DAT, mixed DAT/vascular dementia, Lewy body dementia, or dementia of PD, and memantine is approved for DAT only [20]. However, dementia medications are widely used off-label due the lack of therapies for other types of dementia and the difficulty of excluding the presence of an Alzheimer's component of dementia [21].

### 4.1. Strengths and Weaknesses

Our study has a number of strengths, including its large size and well-defined population. Several limitations must also be considered. The VAHCS is not a closed system, and it is impossible to accurately identify patients who may have outside providers, complicating the interpretation of coding and resource utilization in our dementia population. Diagnoses in this study are based on ICD-9 coding and have not been validated by record review; therefore, there may be misclassification of dementia. Our study is limited to the New England region, and prior research has shown that the prevalence of dementia varies by VISN location [2]. Finally, our population is limited to the veteran population, and generalizability to other populations is uncertain.

## 5. Conclusion

This study presents an overview of current diagnostic and treatment patterns for AD in a large veterans population. The delays in recognition of dementia, insufficient diagnostic assessment, and inappropriate use of dementia medication in this study are not specific to the VA but remain important targets for quality improvement. Additional medical record review is needed in order to demonstrate the validity of recorded dementia diagnoses, document the adequacy of dementia workups, and estimate the level of cognitive impairment of newly diagnosed patients with dementia. Finally, a study of dementia using the national VA database would generate important information about regional variations in dementia care.

## Figures and Tables

**Table 1:** Initial dementia diagnoses by provider type.

Diagnosis by	Dementia, *N* = 7,356			Cognitive impairment, *N* = 1,643				Total
	AD	VD	DNOS	MCI*	Memory loss	CD-CVD	CDNOS	
	*N* (%)	*N* (%)	*N* (%)	*N* (%)	*N* (%)	*N* (%)	*N* (%)	
PCP	1519 (67.6)	1083 (80.8)	2409 (63.9)	17 (37.8)	763 (71.2)	11 (28.2)	186 (38.1)	**5988 (66.5)**
Mental health (psychology/psychiatry)	147 (6.5)	67 (5.0)	410 (10.9)	9 (20.0)	28 (2.6)	2 (5.1)	75 (15.4)	**738 (8.2)**
Neuropsychology	35 (1.6)	31 (2.3)	325 (8.6)	1 (2.2)	8 (0.7)	1 (2.6)	49 (10.0)	**450 (5.0)**
Geriatrician	90 (5.9)	33 (2.5)	99 (2.6)	3 (6.6)	35 (3.2)	9 (23.1)	31 (6.4)	**300 (3.3)**
Neurologist	245 (10.0)	40 (3.0)	166 (4.4)	7 (15.6)	150 (14.2)	10 (25.6)	71 (14.5)	**689 (7.7)**
Other providers	211 (8.4)	86 (6.4)	360 (9.6)	8 (17.8)	87 (8.1)	6 (15.4)	76 (15.6)	**834 (9.3)**
Total	**2247 (100)**	**1340 (100)**	**3769 (100)**	**45 (100)**	**1071 (100)**	**39 (100)**	**488 (100)**	**8999 (100)**

DAT: Alzheimer's type dementia; VD: vascular dementia; DNOS: dementia not otherwise specified; MCI: mild cognitive impairment; CD-CVD: late cognitive effects of cerebrovascular disease; CDNOS: cognitive disorder not otherwise specified.
